# Whole Genome Analysis of Human Rotaviruses Reveals Single Gene Reassortant Rotavirus Strains in Zambia

**DOI:** 10.3390/v13091872

**Published:** 2021-09-18

**Authors:** Wairimu M. Maringa, Julia Simwaka, Peter N. Mwangi, Evans M. Mpabalwani, Jason M. Mwenda, M. Jeffrey Mphahlele, Mapaseka L. Seheri, Martin M. Nyaga

**Affiliations:** 1 Next Generation Sequencing Unit, Division of Virology, Faculty of Health Sciences, University of the Free State, Bloemfontein 9300, South Africa; makena96wairimu@gmail.com (W.M.M.); MwangiPN@ufs.ac.za (P.N.M.); 2 Virology Laboratory, Department of Pathology and Microbiology, University Teaching Hospital, Adult and Emergency Hospital, Lusaka 10101, Zambia; juliachibumbya@gmail.com; 3 Department of Paediatrics and Child Health, School of Medicine, University of Zambia, Ridgeway, Lusaka RW50000, Zambia; evans.mpabalwani@unza.zm; 4 World Health Organization, Regional Office for Africa, Brazzaville P.O. Box 06, Congo; mwendaj@who.int; 5 Office of the Deputy Vice Chancellor for Research and Innovation, The North-West University, Potchefstroom 2351, South Africa; jeffrey.mphahlele@nwu.ac.za; 6 Diarrhoeal Pathogens Research Unit, Faculty of Health Sciences, Sefako Makgatho Health Sciences University, Pretoria 0204, South Africa; mapaseka.seheri@smu.ac.za

**Keywords:** rotavirus, whole genome, genotype constellation, intergenogroup reassortment, amino acid, Rotarix^®^

## Abstract

Rotarix^®^ vaccine was implemented nationwide in Zambia in 2013. In this study, four unusual strains collected in the post-vaccine period were subjected to whole genome sequencing and analysis. The four strains possessed atypical genotype constellations, with at least one reassortant genome segment within the constellation. One of the strains (UFS-NGS-MRC-DPRU4749) was genetically and phylogenetically distinct in the VP4 and VP1 gene segments. Pairwise analyses demonstrated several amino acid disparities in the VP4 antigenic sites of this strain compared to that of Rotarix^®^. Although the impact of these amino acid disparities remains to be determined, this study adds to our understanding of the whole genomes of reassortant strains circulating in Zambia following Rotarix^®^ vaccine introduction.

## 1. Introduction

Group A rotavirus (RVA), a widespread and infectious pathogen that causes dehydrating diarrhea, particularly in children under five years of age, was estimated to have caused approximately 128,000 deaths in 2016. A greater percentage of these deaths (approximately 105,000) occurred in sub-Saharan Africa [1]. The significance of RVA burden of disease led to the development of prophylactic vaccines. In that regard, the World Health Organization (WHO) recommended the use of rotavirus vaccines globally [2]. Four WHO-prequalified rotavirus vaccines (Rotarix^®^, RotaTeq^®^, ROTAVAC^®^, and ROTASIIL^®^) are currently in use in 110 countries worldwide as of 5 April 2021 [3]. The two-dose monovalent vaccine Rotarix^®^ (RV1; GlaxoSmithKline Biologicals, Belgium) consists of a single human G1P[8] strain [2]. In sub-Saharan Africa, Rotarix^®^ is used in countries such as Kenya, Mauritania, Namibia, Niger, and Zimbabwe [3]. This vaccine was introduced in Lusaka, Zambia in 2012 as a pilot project and then rolled out nation-wide in 2013 [4,5]. Vaccine coverage in Zambia in 2019 was at 90% [6].

Rotaviruses contain 11 segments of double-stranded RNA (dsRNA) that encodes six structural viral proteins (VP1-VP4, VP6-VP7) and five and/or six non-structural proteins (NSP1-NSP5/6) [7]. A mature RVA particle comprises an inner core (VP2), which is surrounded by VP1 and VP3, a middle layer (VP6) and an outer layer (VP7) with spikes of the VP4 protruding from the outer layer [7]. The antigenicity of the outer proteins, VP7 and VP4, is used to classify RVA into G-types (glycoprotein) and P-types (protease-sensitive), respectively [7]. Since they are targets of neutralizing antibodies that may provide serotype-specific and/or cross-protective immunity, these two proteins are considered critical for vaccine development [8]. Further, a whole genome based genotyping system was established by the Rotavirus Classification Working Group (RCWG), whereby specific genotypes are assigned to the 11 segments of RVA. This system established three human RVA genogroups exhibiting the Wa-like (G1-P[8]-I1-R1-C1-M1-A1-N1-T1-E1-H1), DS-1-like (G2-P[4]-I2-R2-C2-M2-A2-N2-T2-E2-H2), or the AU-1-like (G3-P[9]-I3-R3-C3-M3-A3-N3-T3-E3-H3) constellations [9,10].

Due to the segmented genome of RVA, it is common for reassortment events to occur, which play a key role in generating the genetic diversity of the virus [11]. It is crucial to understand genetic exchange through reassortment, particularly those belonging to the two major genogroups, as well as various evolutionary mechanisms that contribute to genetic diversity. RVA genomes have high rates of mutation and are subject to frequent reassortment events, which are primarily responsible for rotavirus evolution [11,12,13,14,15,16]. RVA with unusual G-P combinations such as G1P[4], G2P[6], G2P[8], G3P[4], and G8P[4] are known to circulate in human populations as a result of intergenogroup reassortment between co-circulating strains. The G1P[4] and G2P[8] have been shown to circulate among G1P[8] and G2P[4] strains [16,17,18,19,20,21]. Most human RVA strains possess either a typical Wa-like or DS-1-like constellation and are thought to have an evolutionary fitness advantage that allows them to spread widely and persist in human populations [22,23]. Nevertheless, after the isolation of two naturally occurring intergenogroup reassortants between Wa-like and DS-1-like in Bangladesh in 1985–1986 [24], RVA strains possessing mixed gene constellations of human and/or animal origin have been documented in various parts of the world [25,26,27,28,29,30,31,32,33,34].

RVA strain surveillance based on conventional genotyping of VP7 and VP4 has been conducted in Zambia [35]. Unusual G- and P- combinations such as G1P[6] and G9P[6] were reported in 2011 before Rotarix^®^ was implemented. On the contrary, only the G2P[6] was reported post-vaccine implementation [35]. However, there is a dearth of Zambian whole genome sequence data. Here we report the whole genomes of four intergenogroup reassortant strains identified between 2014 and 2016 during the ongoing RVA surveillance in Zambia, to understand the mechanisms that result in genetic diversity among Zambian RVA post-Rotarix^®^ introduction.

## 2. Materials and Methods

### 2.1. Ethics Statement

Ethical approval was obtained from the Health Sciences Research Ethics Committee (HSREC) at the University of the Free State, Bloemfontein, South Africa (Ethics number UFS-HSD2020/0277/2104).

### 2.2. Study Samples

Stool samples were collected in the post-vaccine period from children who presented with acute gastroenteritis. The demographics and clinical profiles of the children from whom the study samples were taken at Arthur Davidson Children’s Hospital (ACDH) in Ndola and University Teaching Hospital (UTH) in Lusaka are shown in Table 1. 

The four Zambian strains analyzed in this study were collected from one female and three male children aged between 5–20 months, as part of the ongoing rotavirus surveillance by the WHO/AFRO. The strains demonstrated a sporadic transmission pattern, devoid of any sign of an outbreak infection, as they were identified in different parts of Ndola and Lusaka. Further, clinical information indicated that all the children had diarrhea that lasted between a day and four days, with varying frequencies. Similarly, three children presented with two to three days of intermittent vomiting, and two children presented with fever. There were two cases of severe dehydration and one case of moderate dehydration because of diarrhea and vomiting. Two of the four children had been vaccinated, while the other two were not vaccinated. However, all the strains were detected post-Rotarix^®^ vaccine implementation (2014–2016). No mortality resulted due to illness, as all children fully recovered.

Thereafter, the samples were screened for presence of RVA antigen using the ProSpecT™ Rotavirus Microplate Assay Kit (Oxoid limited, Basingstoke, Hampshire, UK) at the Virology laboratory in Lusaka, Zambia. The samples were then sent to the Diarrhoeal Pathogens Research unit (DPRU), a WHO Regional Reference Laboratory (Pretoria, South Africa) as part of the annual rotavirus surveillance conducted by the WHO-Regional Office for Africa. The samples were first conventionally genotyped at the DPRU before being shipped to the University of the Free State, Next Generation Sequencing unit (UFS-NGS) for whole genome sequencing and analysis. Four RVA strains from a total of 133 were seen to be reassortant strains after whole genome analysis which formed the basis of this study. To address two Terms of Reference between WHO and UFS-NGS, phylogenetic analysis was performed on all strains, and the possible antigenic disparities between Rotarix^®^, global reference strains and one distinct study strain was assessed.

### 2.3. Extraction of Double-Stranded RNA and cDNA Synthesis

Viral double-stranded RNA was extracted and purified using a MinElute^®^ gel extraction kit (Qiagen, Hilden, Germany) according to already established methods [33,36]. Thereafter, the integrity of purified RNA was determined by electrophoresis on 1% TBE agarose gel stained with ethidium bromide (Sigma-Aldrich, Saint Louis, MO, USA). Before proceeding with synthesis of complementary DNA (cDNA), the purified dsRNA was first quantified on a Biodrop µLite platform (Biochrom, Cambridge, UK). Samples with a 1.8–2.2 absorbance ratio were considered pure for further processing [37]. A Maxima H Minus Double Stranded cDNA kit (Thermo Fisher Scientific, Waltham, MA, USA) was used in the synthesis of cDNA, according to manufacturer’s instructions with minor modifications. The variations were captured in the UFS-NGS SOP as follows: denaturation of dsRNA was performed at 95 °C for five minutes and first strand synthesis was carried out at 50 °C for two hours. The generated cDNA was later purified using an MSB^®^ Spin PCRapace purification kit (Stratec, Invitek molecular, Berlin, Germany).

### 2.4. DNA Library Preparation and Illumina^®^ Sequencing

A Nextera^®^ XT DNA library preparation kit (Illumina^®^, San Diego, CA, USA) was utilized to construct a DNA library according to the manufacturer’s instructions. This process involved fragmenting DNA and subsequent addition of dual barcodes to the DNA fragments. Agencourt AMPure magnetic beads (Beckman Coulter, Indianapolis, Indiana, USA) were used to purify the barcoded libraries while simultaneously selecting for an average insert of 300 bp (range 200–400 bp). Subsequently, the libraries were validated and quantified prior to sequencing on a 2100 Bioanalyzer platform (Agilent Technologies, Santa Clara, CA, USA) and the Qubit^™^ 3.0 fluorometer (Invitrogen, Carlsbad, CA, USA), respectively. The validated and quantified libraries were pooled and whole genome sequencing was performed on an Illumina^®^ MiSeq platform using a V3 MiSeq reagent kit (Illumina^®^, San Diego, CA, USA) for 600 cycles to generate 2 × 301 bp paired reads. A 5% PhiX DNA control spike-in was used.

### 2.5. Genome Assembly

Sequence reads obtained from the Illumina^®^ MiSeq platform in FASTQ format were first trimmed and subsequently assembled using Geneious^®^ Prime 2019.2.1 (https://www.geneious.com/; accessed 5 April 2021) [38]. Genome assembly comprised both reference mapping as well as de novo assembly.

### 2.6. Identification of Genotype Constellations

The genotype of each of the 11 genome segments of the four Zambian RVA strains were identified on the Virus Pathogen Resource (ViPR), an online bioinformatics database and analysis resource for virological research [39]. The Basic Local Alignment Search Tool (BLAST) was also utilized as a complementary tool for genotype identification [40].

### 2.7. Phylogenetic Analysis

Reference sequences were compiled using BLAST as well as the Virus Variation Resource hosted by the National Centre for Biotechnology Information (NCBI) [40,41]. Multiple alignments were made for each gene using the MAFFT plugin in Geneious^®^ Prime version 2019.2.1 (https://www.geneious.com/; accessed 5 April 2021) and MUSCLE algorithm that is present in MEGA 6 [38,42,43]. Pairwise nucleotide and amino acid sequence identity matrices were calculated using the *p*-distance algorithm in MEGA 6 [43]. A maximum likelihood tree was constructed for each genome segment. Substitution models that best fit the data were selected based on corrected Akaike Information Criterion (AICc) in MEGA 6 [44]. The models used in this study were: GTR+G+I (VP1), TN93+G (VP2), GTR+I (VP3 and NSP1), T92+G+I (VP4 and VP7), T92+G (VP6, NSP2, NSP4, and NSP5), and TN93+I (NSP3). Branch support was estimated with 1000 bootstrap replicates [45].

### 2.8. Protein Modelling

Protein modelling was performed using the SWISS MODEL online server (SWISS-MODEL (expasy.org)) [46,47]. The RVA spike protein databank structure, 2dwr.1, was selected from the SWISS MODEL template library and had an X-ray diffraction resolution value of 2.50 Å. The stereochemical quality of the protein structure was assessed using the Structure Assessment feature in SWISS MODEL. The protein structure was modified and visualised using PyMOL (http://www.pymol.org/; accessed 5 April 2021) [48].

## 3. Results

### 3.1. Genotyping Based on Whole Genome Constellations

Following Illumina^®^ MiSeq sequencing, complete or nearly complete nucleotide sequences for each of the 11 genes of the four study strains were obtained. The contig lengths and number of reads after assembly are shown in Table 2. The strains were named as RVA/Human-wt/ZMB/UFS-NGS-MRC-DPRU13232/2016/G1P[8], RVA/Human-wt/ZMB/UFS-NGS-MRC-DPRU13541/2016/G1P[8], RVA/Human-wt/ZMB/UFS-NGS-MRC-DPRU13327/2016/G2P[4], and RVA/Human-wt/ZMB/UFS-NGS-MRC-DPRU4749/2014/G2P[8] according to the guidelines for the uniformity of RVA by the RCWG, henceforth referred to as UFS-NGS-MRC-DPRU13232, UFS-NGS-MRC-DPRU13541, UFS-NGS-MRC-DPRU13327, and UFS-NGS-MRC-DPRU4749, respectively.

The genotype constellations demonstrated that the strains were generated through reassortment between Wa-like and DS-1-like strains. Applying the whole genome-based genotyping system [9,10], UFS-NGS-MRC-DPRU13232, UFS-NGS-MRC-DPRU13541, UFS-NGS-MRC-DPRU13327, and UFS-NGS-MRC-DPRU4749 had the following constellations: G1-P[8]-I1-R1-C1-M1-A1-**N2**-T1-E1-H1, G1-P[8]-I1-R1-C1-M1-A1-**N2**-T1-E1-H1, G2-P[4]-I2-R2-C2-M2-A2-**N1**-T2-E2-H2 and G2-**P**[8]-I2-R2-C2-M2-A2-N2-T2-E-H2, respectively (Table 2) and were therefore considered mono-reassortants, as shown on the bolded genotypes. Strain UFS-NGS-MRC-DPRU13232 and UFS-NGS-MRC-DPRU13541 possessed Wa-like constellations except for the N2 NSP2 genotype. Strain UFS-NGS-MRC-DPRU13327 and UFS-NGS-MRC-DPRU4749 possessed DS-1-like constellations with the exception of N1 NSP2 genotype and P[8] VP4 genotype, respectively.

The 11 genes of the two Wa-like Zambian strains (UFS-NGS-MRC-DPRU13232 and UFS-NGS-MRC-DPRU13541) exhibited a high level of sequence conservation with >99% sequence identity to each other. On the other hand, the two DS-1-like Zambian strains (UFS-NGS-MRC-DPRU13327 and UFS-NGS-MRC-DPRU4749) exhibited high sequence identity (>97%) in the VP7, VP6, VP2, NSP1, NSP3, and NSP5 genes, whereas lower identities were observed in the VP4, VP1, VP3, NSP2, and NSP4 genes (82.7%, 91.0%, 87.9%, 82.7%, and 90.9%, respectively) (Supplementary data 1).

### 3.2. Phylogenetic and Sequence Analysis

To understand the genetic relationship of the four Zambian strains with global stains, a phylogenetic tree was resolved for each of the 11 gene segments. For the designation of lineages in the VP7, VP4, and VP1 trees, closely related strains as well as strains on the respective lineages, were selected from the GenBank using previously published articles as reference [49,50,51,52,53,54].

#### 3.2.1. Phylogenetic Analysis of the VP7 Genes (G1 and G2)

**Figure 1:**
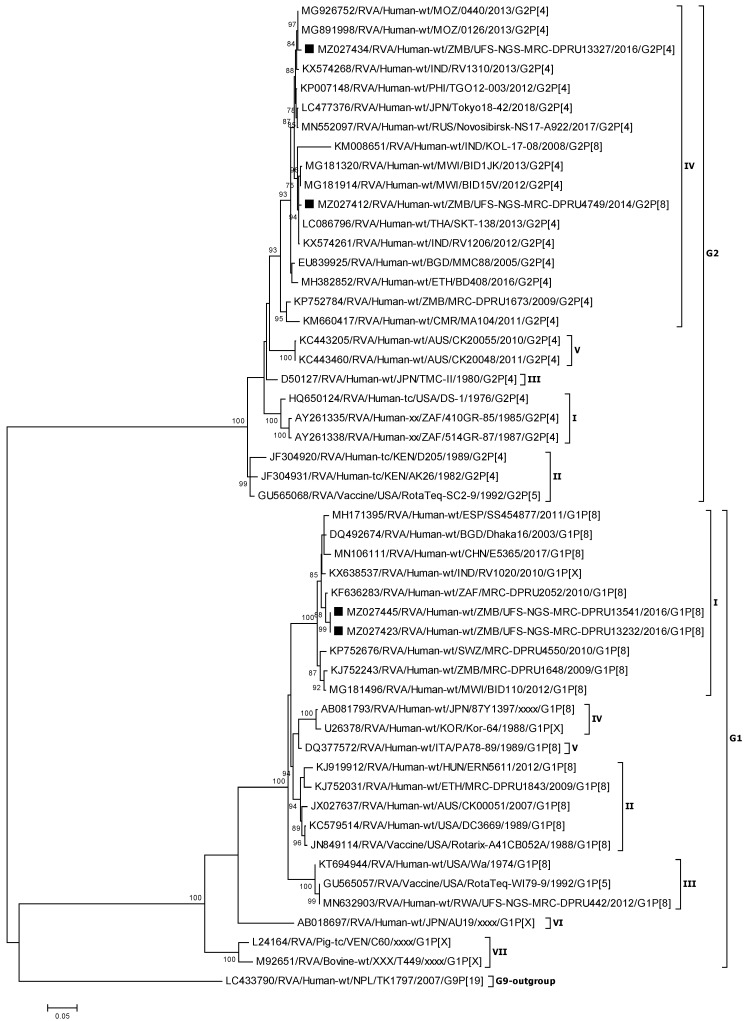
VP7 phylogenetic tree of the Zambian G1 and G2 strains indicated by black squares along with representative strains. Phylogenetic analysis was conducted using the maximum likelihood method with bootstrap values of 1000 replicates. The scale at the bottom indicates the number of nucleotide substitutions per site. Percent values of bootstrap values greater than or equal to 70 is indicated on the branch nodes.

Reference RVA strains utilized in this analysis segregated into the known seven G1 lineages and five G2 lineages [51,52]. A multiple sequence alignment and phylogenetic analysis of the VP7 genes of the four study strains showed that the Zambian G1 strains (UFS-NGS-MRC-DPRU13232 and UFS-NGS-MRC-DPRU13541) clustered with other reference strains in lineage G1 I (Figure 1). Lineage G1 I was comprised of African and Asian strains identified between 2003–2017 with maximum nucleotide (nt) and amino acid (aa) identities ranging from 96.8–99.1% and 97.5–99.7% with the two Zambian G1 strains (Figure 1; Supplementary data 1). Among the two Zambian G1 strains, the nt and aa identity was 100%. On the other hand, the Zambian G2 strains (UFS-NGS-MRC-DPRU4749 and UFS-NGS-MRC-DPRU13327 clustered in lineage G2 IV along with strains from Asia and Africa with nt (aa) identities of 93.8–99.6% (92.6–100%) (Figure 1; Supplementary data 1). A nt and aa similarity of 97.8% and 98.5% was shared between the two Zambian G2 strains (Supplementary data 1).

#### 3.2.2. Phylogenetic Analysis of the VP4 Genes (P[4] and P[8])

**Figure 2:**
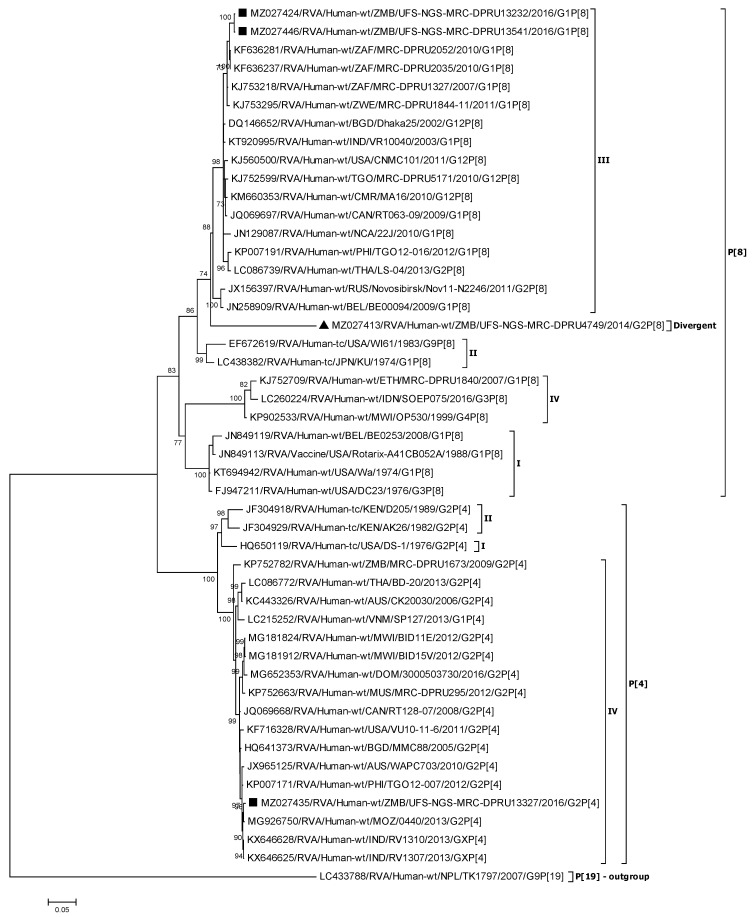
VP4 phylogenetic tree of the Zambian P[4] and P[8] strains indicated by black squares along with representative strains. Strain UFS-NGS-MRC-DPRU4749, indicated by a black triangle, is a divergent strain. Phylogenetic analysis was conducted using the maximum likelihood method with bootstrap values of 1000 replicates. The scale at the bottom indicates the number of nucleotide substitutions per site. Percent values of bootstrap values greater than or equal to 70 is indicated on the branch nodes.

The VP4 P[8] and P[4] Zambian strains were compared to global selected reference strains that belong to the already established four P[4] and four P[8] lineages [51,53]. Based on the VP4 phylogenetic tree, two of the Zambian P[8] strains (UFS-NGS-MRC-DPRU13232, and UFS-NGS-MRC-DPRU13541) co-clustered in lineage P[8] III and shared nt (aa) identity of 99.8% and 99.9%, respectively (Figure 2; Supplementary data 1). Lineage P[8] III consisted of predominantly African strains (Cameroon, Togo, South Africa, and Zimbabwe) that showed nt (aa) identities of 97.6–99.0% (98.7–99.2%) to the two Zambian P[8] strains. The vaccine strain, RVA/Vaccine/USA/Rotarix-A41CB052A/1988/G1P[8], clustered in lineage P[8] I with nt (aa) identities of 90.3–90.4% (93.9–94.1%) to the two aforementioned Zambian P[8] strains.

Interestingly, UFS-NGS-MRC-DPRU4749 clustered separately from the other lineages, including that containing Rotarix^®^ (Lineage P[8] I; nt 84.9%), as well as the most common lineage globally (Lineage P[8] III) [55] that contained the other Zambian P[8] strains (Figure 2; Supplementary data 1). This strain was closest to a South African strain that clustered in lineage P[8] III, RVA/Human-wt/ZAF/MRC-DPRU2035/2010/G1P[8] with nt (aa) identity of 90.2% (92.8%) (Supplementary data 1).

For the P[4] Zambian strain, UFS-NGS-MRC-DPRU13327 clustered with lineage P[4] IV strains and exhibited maximum nt (aa) similarity of 99.6% (99.2%) and 99.4% (99.1%) to a Mozambican strain and an Indian strain, respectively.

#### 3.2.3. Comparison of the VP4 Antigenic Epitopes of Zambian G2P[8] to Rotarix^®^

**Figure 3:**
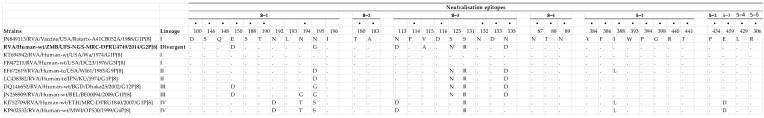
Alignment of the VP4 antigenic epitopes of the divergent study strain, UFS-NGS-MRC-DPRU4749 that is highlighted in bold, along with global P[8] strains belonging to the already defined four different P[8] lineages, in relation to Rotarix^®^. Antigenic epitopes are divided into two subunits: VP8* (8-1 to 8-4) and VP5* (5-1 to 5-5). The bold black dots (•) indicate amino acid changes in the residues that have been shown to escape neutralisation with monoclonal antibodies. The normal dots (.) represent conserved amino acids relative to Rotarix^®^.

The pattern of aa substitution occurring in P[8] strains in each lineage, including the phylogenetically distinct Zambian strain UFS-NGS-MRC-DPRU4749 that was seen to be phylogenetically distinct, was analyzed relative to that of Rotarix^®^. It was observed that there were 26 fully conserved aa residues. Overall, most of the aa changes in the P[8] strains relative to Rotarix^®^ were displayed in the VP8* (8-1 and 8-3) region (Figure 3). Lineage I P[8] strains possessed the same aa at all positions. While lineage II P[8] strains had five aa substitutions from Rotarix^®^ (N195D, S125N, S131R, N135D, and I388L), the I388L substitution occurred only in one of the lineage II P[8] strains. Lineage III P[8] strains had six aa substitutions (E150D, N194G, N195G, S125N, S131R, and N135D) relative to Rotarix^®^. However, the substitution N195G was present in only one of the two lineage III P[8] strains. Lineage IV P[8] strains also known as *OP354-like* [56,57,58] had seven aa changes (N192D, N194T, N195S, N113D, S131R, I388L, and E459D).

**Figure 4:**
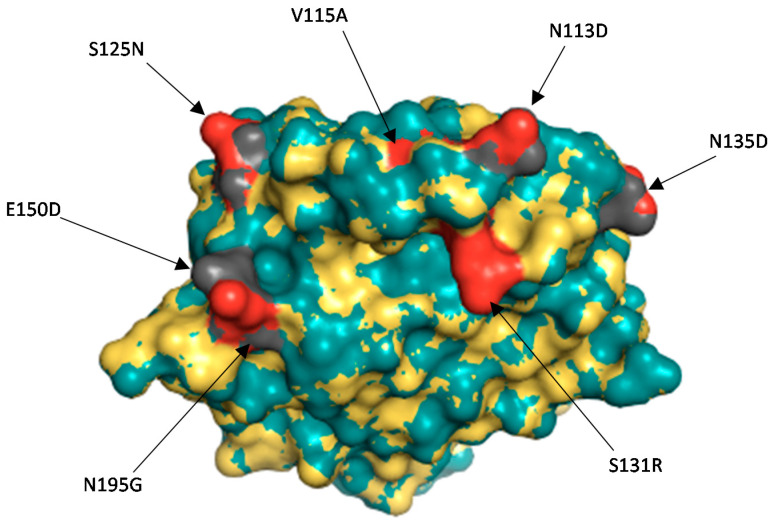
Surface representation of the VP8* protein of Rotarix^®^ and the divergent study strain UFS-NGS-MRC-DPRU4749. The superposition of the two structures has the root square mean deviation of 0.048 Å. Rotarix^®^ structure is represented by the teal colour whereas the Zambian P[8] strain is indicated in yellow. The red colour represents the amino acid changes observed on the Zambian study strain as compared to Rotarix^®^ vaccine strain in grey.

Comparison of the divergent Zambian P[8] strain against Rotarix^®^ showed 30 identical aa residues spanning the VP4 antigenic epitopes (Figure 3). Seven aa changes, E150D, N195G, N113D, V115A, S125N, S131R, and N135D, were seen in the study strain relative to Rotarix^®^ (Figure 3). These changes were located on the surface of the protein structure (Figure 4). Analysis of the Zambian P[8] strain relative to two selected strains of the most common lineage, lineage P[8] III [55], identified two aa differences (D113N and A115V).

#### 3.2.4. Phylogenetic Analysis of the VP1 Gene

**Figure 5:**
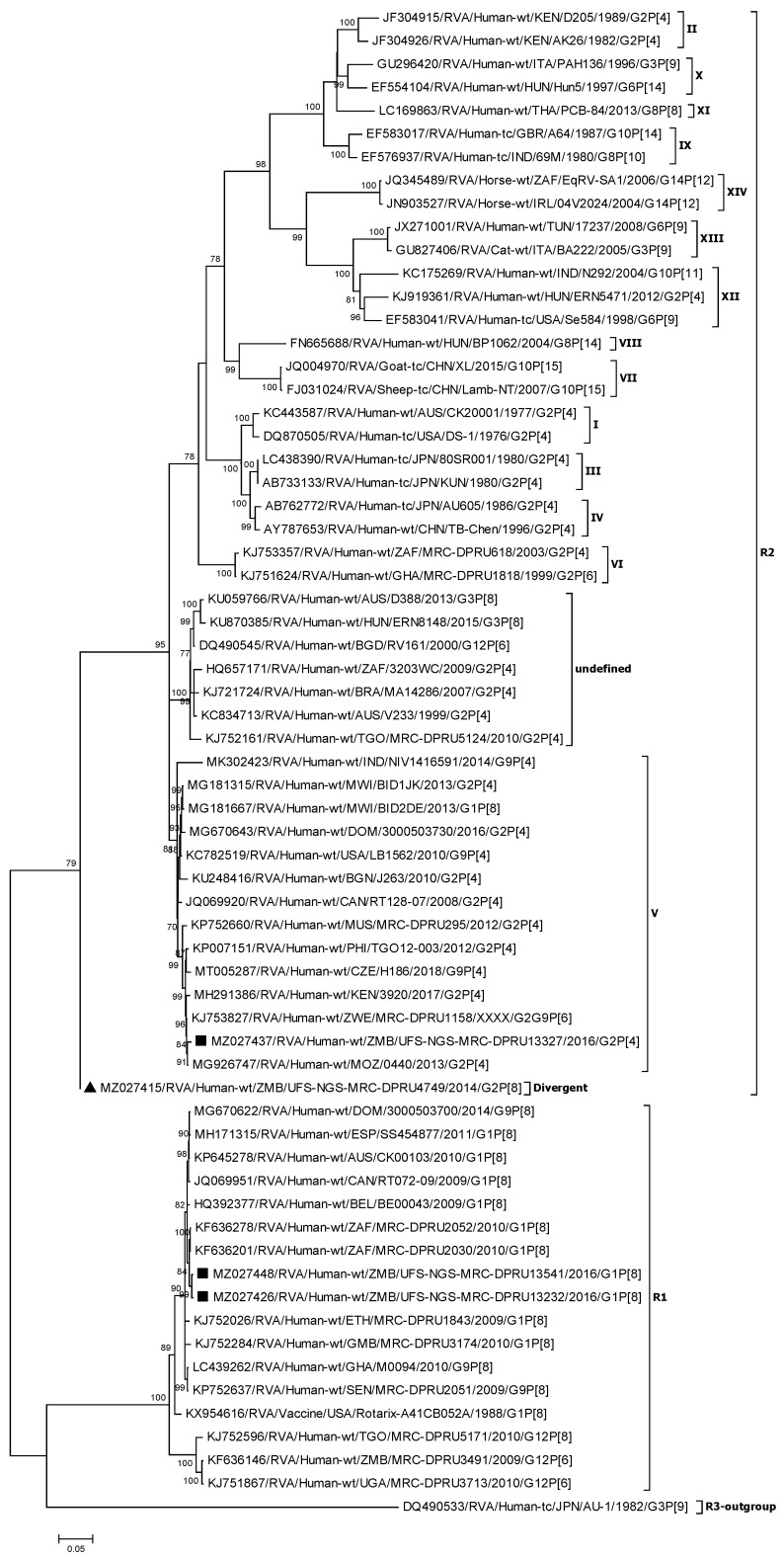
VP1 phylogenetic tree of the Zambian R1 and R2 strains indicated by black squares along with representative Strain UFS-NGS-MRC-DPRU4749, indicated by a black triangle is a divergent strain. Phylogenetic analysis was conducted using the maximum likelihood method with bootstrap values of 1000 replicates. The scale at the bottom indicates the number of nucleotide substitutions per site. Percent values of bootstrap values greater than or equal to 70 is indicated on the branch nodes.

The two Zambian Wa-like strains (UFS-NGS-MRC-DPRU13232 and UFS-NGS-MRC-DPRU13541) clustered among R1 African strains. The two strains shared highest nt (aa) similarity of 99.4% (99.4–99.7%) with South African strains RVA/Human-wt/ZAF/MRC-DPRU2030/2010/G1P[8] and RVA/Human-wt/ZAF/MRC-DPRU2052/2010/G1P[8] (Figure 5; Supplementary data 1).

Doan et al. [52] established five lineages for global R2 strains. More recently, Agbemabiese et al. [49] proposed 14 lineages for R2 strains which included human and animal RVA strains. Based on this, one of the two DS-1-like Zambian strains, UFS-NGS-MRC-DPRU13327, clustered in lineage R2 V that mainly comprised of African strains (Figure 5). This strain displayed maximum nt (aa) identities of 99.4% (99.7%) with strains from Zimbabwe and Mozambique (Supplementary data 1). In the VP1 phylogenetic tree, a cluster of strains within the R2 genotype could not be classified under any lineage according to the established designations [49,52] and were therefore named “undefined”.

Strain UFS-NGS-MRC-DPRU4749 clustered independently (Figure 5), and shared the highest similarity to RVA/Human-wt/IND/NIV1416591/2014/G9P[4] that clustered in Lineage R2 V, with nt (aa) identities of 93% (96.6%) (Supplementary data 1).

#### 3.2.5. Phylogenetic Analysis of the VP6, VP2 and VP3 Genes

The VP6, VP2, and VP3 genes of the four Zambian strains clustered among African strains. The VP6 genes of Wa-like strains UFS-NGS-MRC-DPRU13232 and UFS-NGS-MRC-DPRU13541 displayed maximum nt identities (97.7–98.1%) with the VP6 genes of the South African strain RVA/Human-wt/ZAF/MRC-DPRU2052/2010/G1P[8] (Supplementary data 1). Phylogenetically, the two Wa-like Zambian strains co-clustered in lineage I1 (Supplementary data 2). On the other hand, the DS-1-like Zambian strains (UFS-NGS-MRC-DPRU4749 and UFS-NGS-MRC-DPRU13327) clustered separately under lineage I2 among strains identified in Malawi and Mozambique with nt and aa identities of 99.7–99.9% and 99.7–100% (Supplementary data 1; Supplementary data 2).

The VP2 genes of the two Wa-like Zambian strains co-clustered in lineage C1 and exhibited highest nt identity of 98.7% with the South African strain RVA/Human-wt/ZAF/MRC-DPRU2052/2010/G1P[8] and Zimbabwean strain RVA/Human-wt/ZWE/MRC-DPRU1844-11/2011/G1P[8], whereas the two Zambian DS-1-like strains clustered in lineage C2, exhibiting maximum nt (aa) identities of 98.8–99.5% (99.4–100%) with VP2 genes of Malawian and Mozambican strains (Supplementary data 1; Supplementary data 2). Like the VP2 gene, the VP3 genes of the two Wa-like Zambian strains co-clustered in lineage M1 that consisted predominantly of African strains. Highest nt (aa) identities of 99.1–99.2% (98.8–99.0%) was observed to the Zimbabwean strain RVA/Human-wt/ZWE/MRC-DPRU1844-11/2011/G1P[8] and the South African strain RVA/Human-wt/ZAF/MRC-DPRU2052/2010/G1P[8]. The VP3 genes of the two Zambian DS-1-like strains were in two different clusters within the M2 lineage. UFS-NGS-MRC-DPRU13327 showed highest nt similarity (99.4%) with Mozambican strain RVA/Human-wt/MOZ/0440/2013/G2P[4] whereas the other DS-1-like Zambian strain, UFS-NGS-MRC-DPRU4749, was closest to Malawian strains with nt (aa) identities of 99.3–99.6% (99.0–99.4%) (Supplementary data 1; Supplementary data 2).

#### 3.2.6. Phylogenetic Analysis of the NSP1-NSP5 Genes

Phylogenetically, the NSP1, NSP3, NSP4, NSP4, and NSP5 genes of the two Zambian Wa-like strains (UFS-NGS-MRC-DPRU13232 and UFS-NGS-MRC-DPRU13541) co-clustered in lineages A1, T1, E1 and H1, respectively, whereas the two DS-1-like Zambian strains (UFS-NGS-MRC-DPRU4749 and UFS-NGS-MRC-DPRU13327) clustered distant from each other in lineages A2, T2, E2 and H2 (Supplementary data 2). For the NSP2 gene, UFS-NGS-MRC-DPRU13327 clustered in N1, while UFS-NGS-MRC-DPRU4749, UFS-NGS-MRC-DPRU13232 and UFS-NGS-MRC-DPRU13541 co-clustered in lineage N2 (Supplementary data 2).

The NSP1 and NSP4 genes of UFS-NGS-MRC-DPRU13232 and UFS-NGS-MRC-DPRU13541 fell into clusters predominantly comprised of African strains, and were closest to the strains, RVA/Human-wt/ZWE/MRC-DPRU1844-11/2011/G1P[8] and RVA/Human-wt/MRC-DPRU1544/2010/G1P[8], with nt (aa) identities of 98.0–98.5% (97.1–98.6%) (Supplementary data 1; Supplementary data 2). In contrast, the NSP3 and NSP5 genes of the two Zambian G1P[8] strains displayed the highest nt (99.0–99.1%) and aa (98.5–99.7%) identities to Brazilian strains (Supplementary data 1; Supplementary data 2).

For the DS-1-like Zambian strains, UFS-NGS-MRC-DPRU4749 clustered closely with Malawian strains in the NSP1, NSP3, NSP4, and NSP5 genes, displaying nt (aa) identities of 99.2–99.3% (98.6–98.85), 99.4–99.7% (99.7–100%), 98.9% (99.4%) and, 98.6–99.2% (99.0–99.5%), respectively (Supplementary data 1; Supplementary data 2). UFS-NGS-MRC-DPRU13327, on the other hand, was closest related to Mozambican strain RVA/Human-wt/MOZ/0440/2013/G2P[4] in the NSP1, NSP3, and NSP5 genes with maximum nt (aa) identities of 99.5% (99.4%), 99.6% (99.7%), and 99.7% (99.5%) in those respective genes (Supplementary data 1; Supplementary data 2). For the NSP4 gene, UFS-NGS-MRC-DPRU13327 clustered among Asian strains and exhibited the highest nt (aa) identity of 97.7% (98.9%) to strain RVA/Human-wt/IND/RV1206/2012/G2P[4] (Supplementary data 1; Supplementary data 2).

Based on the NSP2 gene, UFS-NGS-MRC-DPRU13327, UFS-NGS-MRC-DPRU13232 and UFS-NGS-MRC-DPRU13541 were seen to be reassortants. The DS-1-like Zambian strain, UFS-NGS-MRC-DPRU13327, belonged to genotype N1 and clustered among strains from Asia, Oceania, and Europe, with maximum nt (aa) identities of 99.4% (100%) and 99.5% (99.7%) to a Russian and Indian strain, respectively (Supplementary data 1; Supplementary data 2). The two Wa-like Zambian strains, UFS-NGS-MRC-DPRU13232 and UFS-NGS-MRC-DPRU13541, along with the DS-1-like strain UFS-NGS-MRC-DPRU4749 belonged to genotype N2 and displayed highest nt (aa) similarity of 99.4–99.8% (98.7–99.7%) to Malawian strains (Supplementary data 1; Supplementary data 2).

## 4. Discussion

The present study reported on four intergenogroup reassortant strains in Zambia. Whole genome sequencing and analyses demonstrated that the four study strains possessed mixed genotypes in at least one gene segment within the constellation between Wa-like and DS-1-like genogroups, hence were considered intergenogroup reassortant strains. Such reassortant strains have been detected in countries such as Germany, Japan, Lebanon, Malawi, Rwanda, Senegal, South Africa, and Zimbabwe [28,30,34,59,60,61,62,63]. A key observation was made regarding strain UFS-NGS-MRC-DPRU4749. This strain was seen to be phylogenetically distinct in the VP4 gene, as it did not cluster into any of the already defined P[8] lineages [51]. VP4 analysis on BLAST and ViPR showed that UFS-NGS-MRC-DPRU4749 possessed a nucleotide variance of almost 10% to the closest strain. The same observation was made in the VP1 gene, whereby the Zambian strain clustered distinctly from other established R2 lineages proposed by Doan et al. [52] and Agbemabiese et al. [49], with a nucleotide variance of 7% to the closest strain. Further, the divergent Zambian strain was supported by bootstrap values of 88% and 79% at the branching node in the VP4 and VP1 phylogenetic trees, respectively. The large genetic distance to other global strains on both nt and aa level concurred with the distinct clustering seen in the VP4 and VP1 phylogenetic trees, thus strain UFS-NGS-MRC-DPRU4749 can be considered as a divergent strain.

The VP4 spike protein is proteolytically cleaved into VP8* and VP5* subunits by trypsin-like proteases present in the gastrointestinal tract of a host, which in the process activates the rotavirus particle [64,65]. The VP5* enables the penetration of the virus by permeabilizing lipid vesicles during infection, while the VP8* is thought to mediate attachment to the host [66,67,68]. Four (8-1 to 8-4) and five (5-1 to 5-5) epitopes are contained in the VP8* and VP5* subunits, respectively, which are targets for neutralizing monoclonal antibodies [55]. Neutralizing antibodies that target the VP8* neutralize infectivity of the virus by inhibiting attachment, while those directed against VP5* are thought to block membrane penetration [69,70]. The VP4 is involved in several important structural and functional roles such as attachment, penetration, and particle maturation. Due to this, the genetic variability is more restricted in human VP4 RVA as compared to the VP7 [7,58,70]. This characteristic is exploited by the current vaccines, Rotarix^®^ which contains a single human G1P[8] and RotaTeq^®^ that contains G1–G4 and a P[8] genotype [71]. Therefore, while the higher genetic variability in the VP7 may compromise immunity induced by vaccines, the VP4 component of vaccines may compensate when a human is infected with a P[8] strain. In agreement with the observation of low genetic variability in VP4, around 70% (26/37) of the aa residues belonging to the global human P[8] RVA strains, including Zambian strain UFS-NGS-MRC-DPRU4749, were fully conserved when compared to the Rotarix^®^ vaccine strain.

Accumulation of point mutations, along with reassortment and other mechanisms of rotavirus evolution, is a key mechanism that generates genetic diversity in RVA over time [11,12,13,14,15]. Seven aa substitutions were identified in the VP8* (8-1 and 8-3) region when the study strain UFS-NGS-MRC-DPRU4749 was compared against Rotarix^®^. Of the seven, four were seen to be radical in nature (N195G, N113D, S131R, and N135D). With respect to the nature of aa, the N195G substitution resulted in a change in polarity (polar to non-polar) whereas N113D, S131R, and N135D resulted in a change in charge (polar neutral to acidic polar negative, polar neutral to basic polar positive, and polar neutral to acidic polar negative, respectively) [72]. One peculiar aa difference was the V115A which occurred only in the study strain. This mutation is considered conservative because the charge and polarity of the aa remained unchanged. It is therefore unlikely that this change would affect protein structure and hydrophobicity [72,73]. The impact of such a change on rotavirus transmission and vaccine effectiveness remains to be determined.

## 5. Conclusions

This study lends credence to reassortment being a major evolutionary mechanism in RVA. Since the other three Zambian strains were also collected during the post-vaccine period, the discovery of the phylogenetically and genetically divergent Zambian G2P[8] strain was unexpected. Given that this strain was identified in an unvaccinated child, it remains unclear whether the aa mutations present in the VP4 gene would have a negative impact on the effectiveness of the vaccine. Continuous surveillance of circulating RVA, along with whole genome sequencing and analysis is therefore critical in monitoring the impact of such reassortant strains on children, as well as their impact on effectiveness of current vaccine products.

## Figures and Tables

**Table 1:** Table showing the demographics and clinical profiles of the children from which the study samples were obtained.

Sample ID and Year	Hospital	The Child’s Place of Residence	Sex	Age	Presenting Illness Symptoms	Dehydration Status and Treatment Administered	Vaccination Status	Outcome of Illness
UFS-NGS-MRC-DPRU4749/2014	ACDH Ndola	Chifubu	Female	5 months	Diarrhoea for 4 days (4 episodes in 24 h), no vomiting, temperature of 39 °C	Moderate dehydration, treated with ORS	Not vaccinated	Alive
UFS-NGS-MRC-DPRU13232/2016	ACDH Ndola	Kawama	Male	7 months	Diarrhoea for 3 days (6 episodes in 24 h), vomiting for 2 days (4 episodes in 24 h), temperature of 38.2 °C	Severe dehydration, treated with IV fluids	Vaccinated (1 dose)	Alive
UFS-NGS-MRC-DPRU13541/2016	ACDH Ndola	Mwange A	Male	8 months	Diarrhoea for 3 days (8 episodes in 24 h), vomiting for 3 days (3 episodes in 24 h), no fever	Severe dehydration, treated with IV fluids	Not vaccinated	Alive
UFS-NGS-MRC-DPRU13327/2016	UTH Lusaka	Kapata	Male	20 months	Diarrhoea for 1 day (3 episodes in 24 h), vomiting for 3 days, no fever	No dehydration, treated with ORS	Vaccinated (2 doses)	Alive

**Table 2:** The whole genome constellation of the four reassortant study strains detected between 2014 and 2016 (post-vaccine period) in Zambia along with the contig length and the number of reads mapped to each contig.

Strain		VP7	VP4	VP6	VP1	VP2	VP3	NSP1	NSP2	NSP3	NSP4	NSP5
UFS-NGS-MRC-DPRU13232	Genotype	G1	P[8]	I1	R1	C1	M1	A1	N2	T1	E1	H1
	Contig length	1062	2359	1356	3301	2717	2591	1567	1059	1074	750	644
	Reads mapped to contig	21,238	4523	14,997	87,349	52,209	52,222	26,784	53,976	30,125	25,306	21,366
UFS-NGS-MRC-DPRU13541	Genotype	G1	P[8]	I1	R1	C1	M1	A1	N2	T1	E1	H1
	Contig length	1063	2359	1352	3301	2729	2591	1567	1059	1074	750	663
	Reads mapped to contig	33,485	10,936	62,838	108,961	79,014	134,489	80,109	33,007	36,184	34,457	12,638
UFS-NGS-MRC-DPRU4749	Genotype	G2	P[8]	I2	R2	C2	M2	A2	N2	T2	E2	H2
	Contig length	1062	2360	1356	3302	2684	2591	1569	1059	1066	750	815
	Reads mapped to contig	1445	4513	2302	6738	4388	5214	2315	1063	1268	916	471
UFS-NGS-MRC-DPRU13327	Genotype	G2	P[4]	I2	R2	C2	M2	A2	N1	T2	E2	H2
	Contig length	1062	2359	1354	3298	2684	2591	1566	1059	1066	751	798
	Reads mapped to contig	24,446	51,762	23,311	67,839	53,795	60,905	25,147	11,048	20,338	13,618	13,618

The Wa-like genogroup is represented in green, while the DS-1-like genogroup is represented in red.

## Data Availability

The authors confirm that all data relevant to this study is contained in the article and the supplementary materials. Additionally, sequences generated in this study were deposited in the GenBank under accession numbers MZ027412-MZ027455.

## Supplementary Materials

The following are available online at https://www.mdpi.com/article/10.3390/v13091872/s1, Supplementary data 1: Identity matrices (nucleotide and amino acid) among strains for the VP7, VP4, VP6, VP1, VP2, VP3, NSP1, NSP2, NSP3, NSP4 and NSP5 calculated using the p-distance algorithm in MEGA 6. Supplementary data 2: Additional phylograms containing Figure S1 (VP6), Figure S2 (VP2), Figure S3 (VP3), Figure S4 (NSP1), Figure S5 (NSP2), Figure S6 (NSP3), Figure S7 (NSP4), and Figure S8 (NSP5).
