# Supplementary material for: Whole Genome Analysis of Human Rotaviruses Reveals Single Gene Reassortant Rotavirus Strains in Zambia
Source: Viruses. 2021 Sep 18;13(9):1872. doi: 10.3390/v13091872 (PMC8472975; doi:10.3390/v13091872)
Supplement: Supplementary file 1 [file viruses-13-01872-s001.zip › Supplementary data 2.pdf]

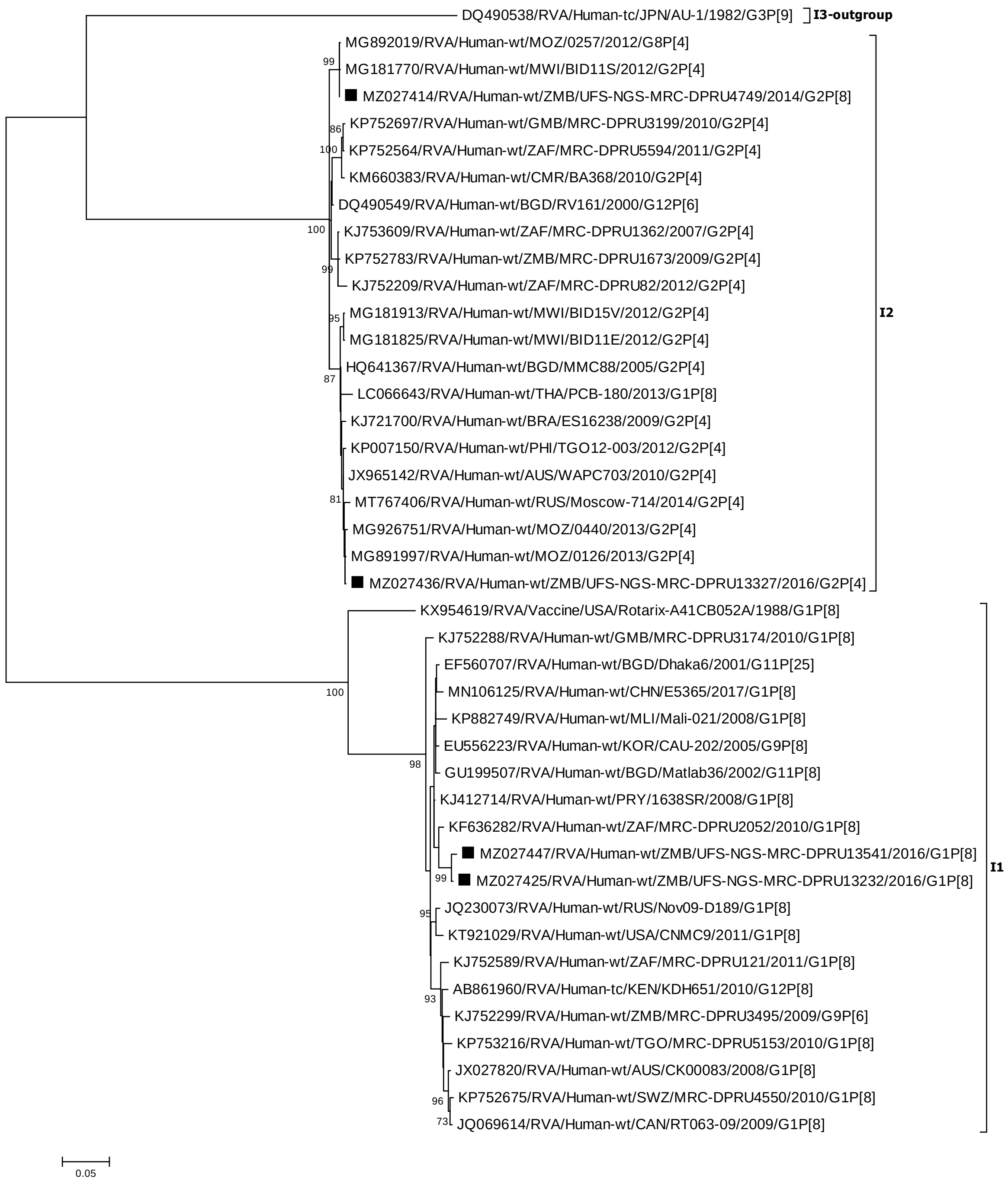

Figure S1. VP6 phylogenetic tree of the four Zambian strains indicated by black squares along with representative strains. Phylogenetic analysis was conducted using the maximum likelihood method with bootstrap values of 1000 replicates. The scale at the bottom indicates the number of nucleotide substitutions per site. Percent values of bootstrap values greater than or equal to 70 is indicated on the branch nodes.

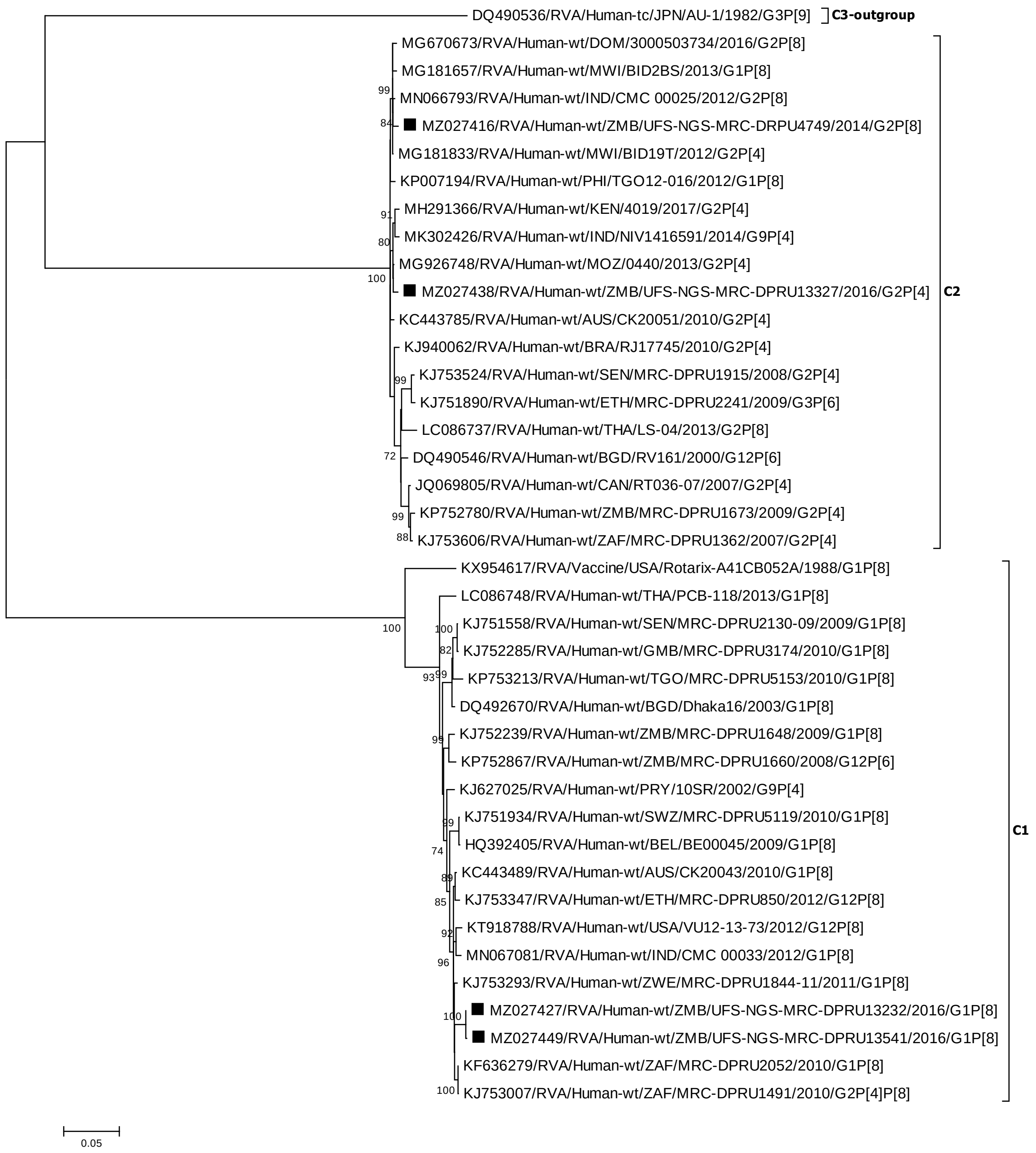

Figure S2. VP2 phylogenetic tree of the four Zambian strains indicated by black squares along with representative strains. Phylogenetic analysis was conducted using the maximum likelihood method with bootstrap values of 1000 replicates. The scale at the bottom indicates the number of nucleotide substitutions per site. Percent values of bootstrap values greater than or equal to 70 is indicated on the branch nodes.

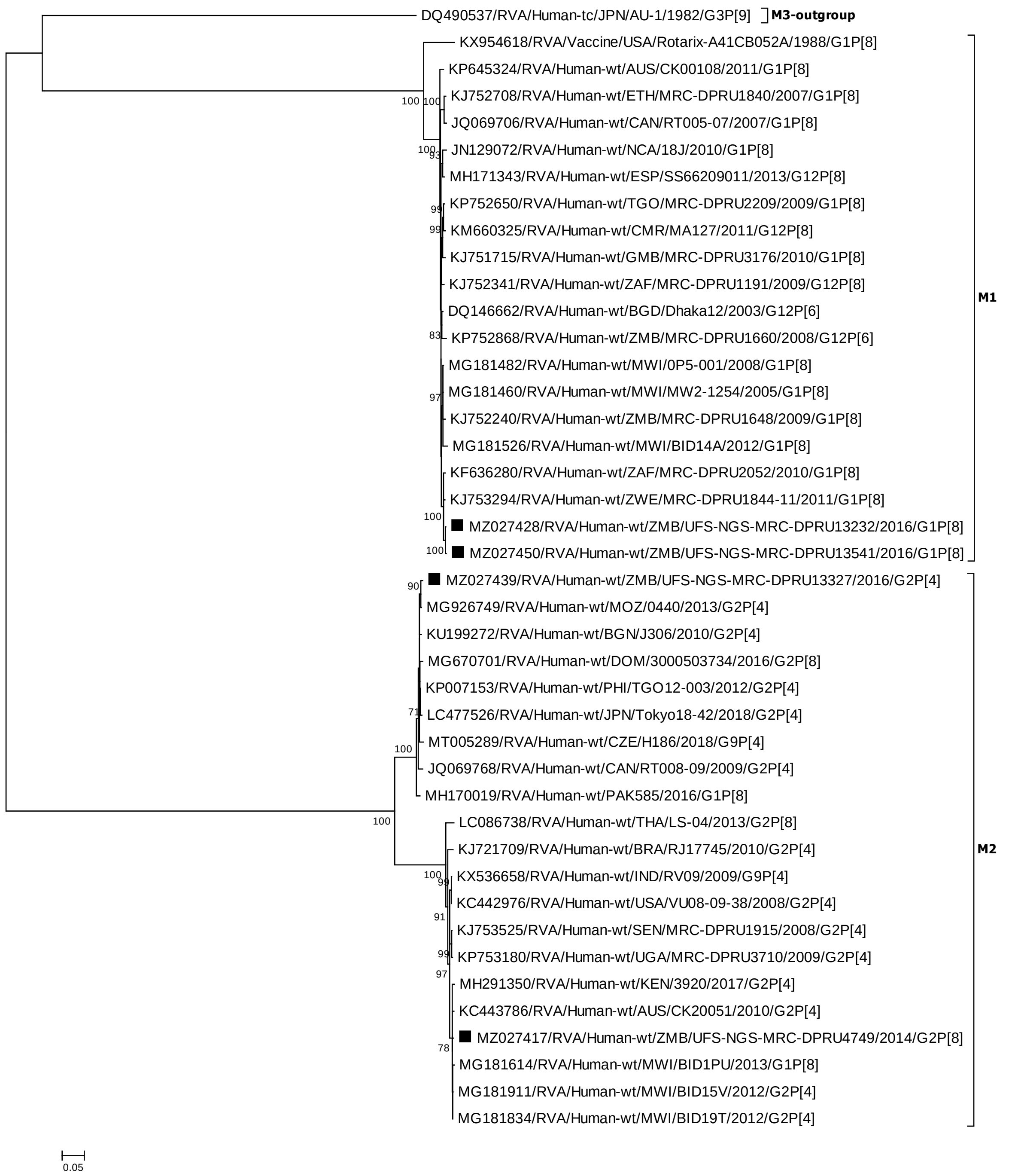

Figure S3. VP3 phylogenetic tree of the four Zambian strains indicated by black squares along with representative strains. Phylogenetic analysis was conducted using the maximum likelihood method with bootstrap values of 1000 replicates. The scale at the bottom indicates the number of nucleotide substitutions per site. Percent values of bootstrap values greater than or equal to 70 is indicated on the branch nodes.

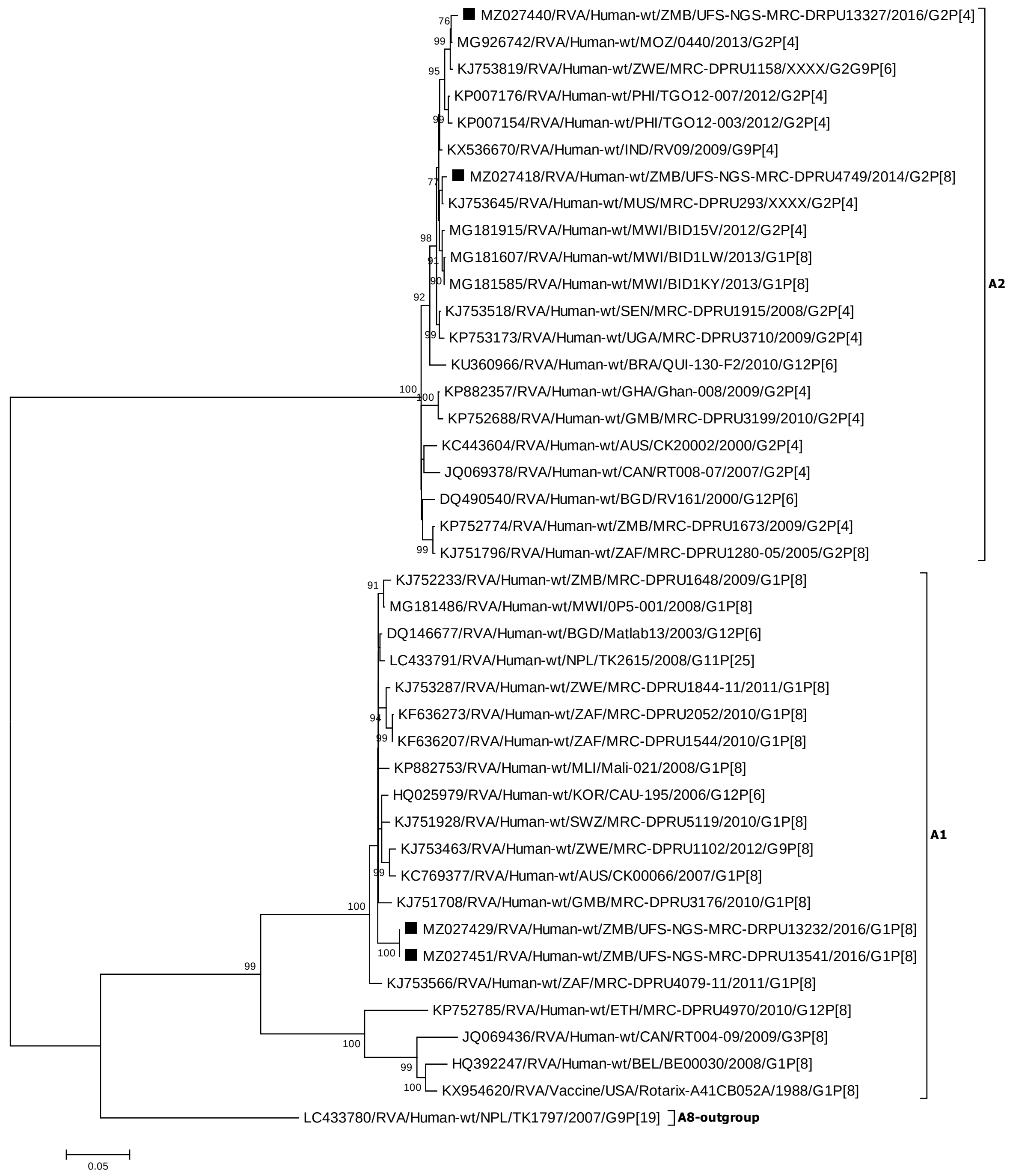

Figure S4. NSP1 phylogenetic tree of the four Zambian strains indicated by black squares along with representative strains. Phylogenetic analysis was conducted using the maximum likelihood method with bootstrap values of 1000 replicates. The scale at the bottom indicates the number of nucleotide substitutions per site. Percent values of bootstrap values greater than or equal to 70 is indicated on the branch nodes.

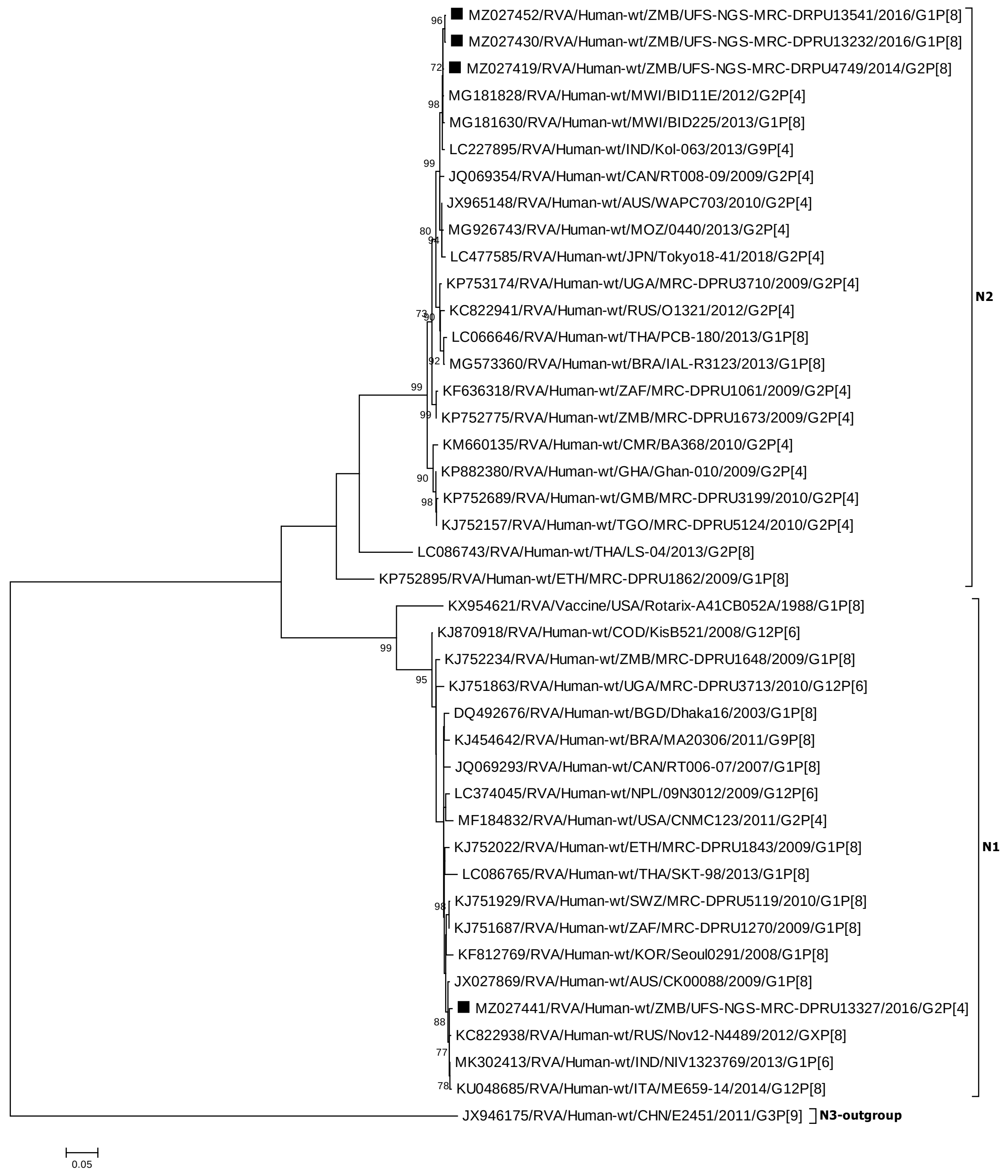

Figure S5. NSP2 phylogenetic tree of the four Zambian strains indicated by black squares along with representative strains. Phylogenetic analysis was conducted using the maximum likelihood method with bootstrap values of 1000 replicates. The scale at the bottom indicates the number of nucleotide substitutions per site. Percent values of bootstrap values greater than or equal to 70 is indicated on the branch nodes.

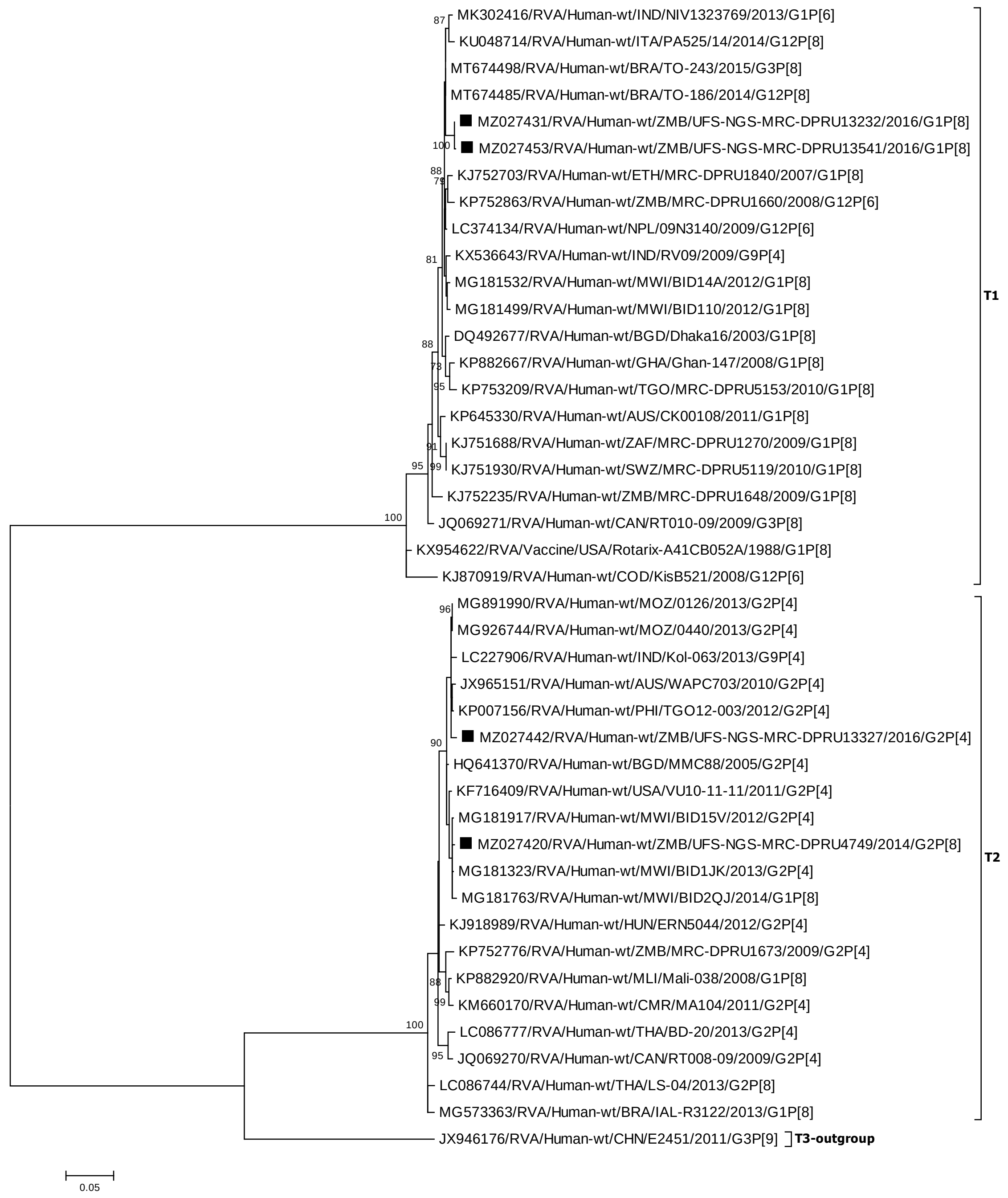

Figure S6. NSP3 phylogenetic tree of the four Zambian strains indicated by black squares along with representative strains. Phylogenetic analysis was conducted using the maximum likelihood method with bootstrap values of 1000 replicates. The scale at the bottom indicates the number of nucleotide substitutions per site. Percent values of bootstrap values greater than or equal to 70 is indicated on the branch nodes.

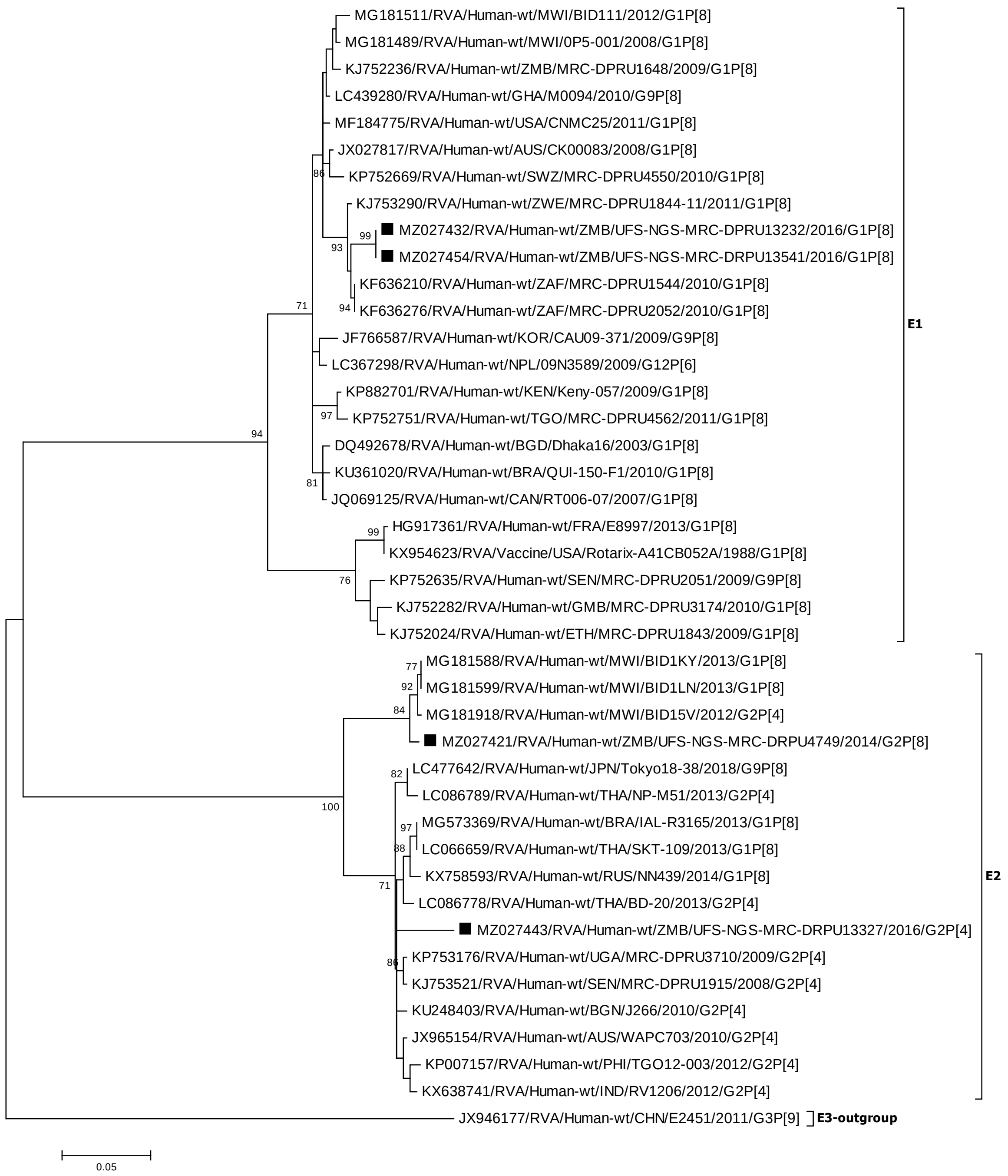

Figure S7. NSP4 phylogenetic tree of the four Zambian strains indicated by black squares along with representative strains. Phylogenetic analysis was conducted using the maximum likelihood method with bootstrap values of 1000 replicates. The scale at the bottom indicates the number of nucleotide substitutions per site. Percent values of bootstrap values greater than or equal to 70 is indicated on the branch nodes.

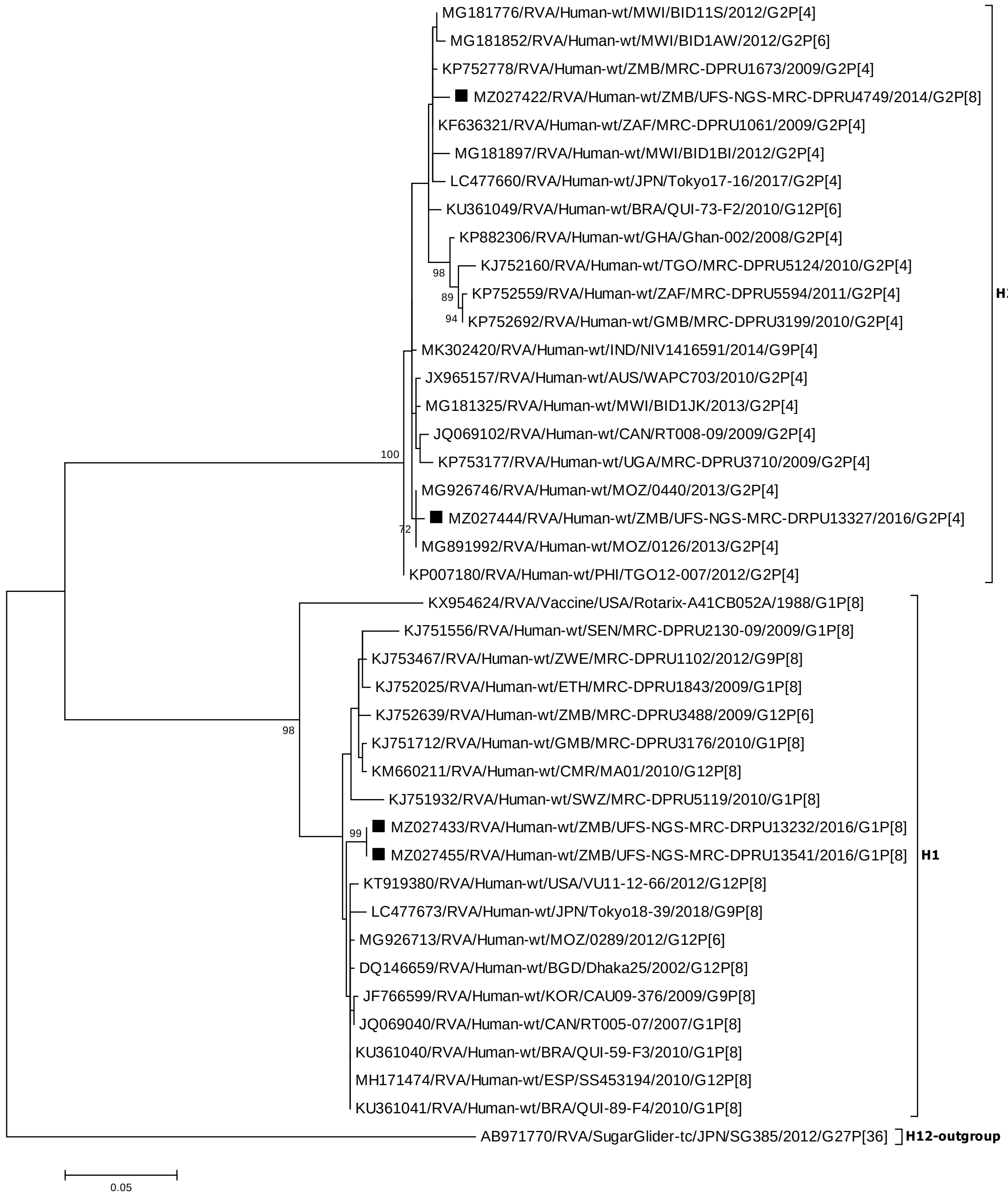

Figure S8. NSP5 phylogenetic tree of the four Zambian strains indicated by black squares along with representative strains. Phylogenetic analysis was conducted using the maximum likelihood method with bootstrap values of 1000 replicates. The scale at the bottom indicates the number of nucleotide substitutions per site. Percent values of bootstrap values greater than or equal to 70 is indicated on the branch nodes.
